# Hemadsorption with Adult CytoSorb® in a Low Weight Pediatric Case

**DOI:** 10.1155/2017/6987167

**Published:** 2017-01-03

**Authors:** Catalin Gabriel Cirstoveanu, Ileana Barascu, Samantha Mc Kenzie Stancu

**Affiliations:** ^1^“Carol Davila” University of Medicine & Pharmacy Bucharest, 37 Dionisie Lupu Street, District 1, 020021 Bucharest, Romania; ^2^“Marie S. Curie Children's Emergency Hospital” Bucharest, Neonatal Intensive Care Unit, 20 Constantin Brancoveanu Street, District 4, 041451 Bucharest, Romania

## Abstract

Cytokine adsorber (CytoSorb) has been used successfully as adjunctive treatment for adult patients with elevated cytokine levels in the setting with severe sepsis and septic shock and to reduce blood myoglobin, unconjugated bilirubin, and conjugated bilirubin. In this article we present the case of a nine-month-old male infant who was admitted to the NICU due to sepsis after cardiac surgery, Fallot tetralogy, and multisystem organ failure (MSOF) including liver failure and renal failure which was successfully treated by a combination of continuous hemodiafiltration (HDF) and hemadsorption with CytoSorb. HDF was safe and effective from the first day for urea removal, but the patient's bilirubin levels kept increasing gradually, culminating on the 9th day with a maximum value of 54 mg/dL of total bilirubin and 31.67 mg/dL of direct bilirubin when we performed hemadsorption with CytoSorb. Over the 49-hour period of hemadsorption, the total bilirubin value decreased from 54 to 14 mg/dL, and the patient's general status improved considerably accompanied by a rapid drop of aminotransferases. Hemodynamic status has been improved as well and inotropes dropped rapidly. The patient's ventilation settings improved during CytoSorb treatment permitting weaning the patient from mechanical ventilation after five days of hemadsorption. The patient was discharged home after 34 days of hospitalization, in a good general status.

## 1. Introduction

Hemadsorption is a means of artificial extracorporeal liver support designed to remove toxins accumulating in patients suffering from acute liver failure (ALF). First developed in the 1950s, although scarcely employed, hemadsorption gained its reputation as an efficacious modality of bridging to liver transplant [1].

In 1996, Bhaduri and Mieli-Vergani proposed the definition of ALF as “a rare multisystem disorder in which severe impairment of liver function, with or without encephalopathy, occurs in association with hepatocellular necrosis in a patient with nonrecognized underlying chronic liver disease” [2].

Although it is a rare entity encountered in neonates, without liver transplantation it is a highly fatal condition, with mortality of 70%. The main parameter used to establish the need for transplantation is an INR ≥ 4, representing a mortality of 90% [3]. The causes of ALF are diverse ranging from perinatal infections and inborn errors of metabolism to septicemia and shock [4, 5]. Due to its markedly high mortality rate, neonates suffering from ALF should be admitted to the Neonatal Intensive Care Unit (NICU) and managed by a highly skilled, multidisciplinary team comprising neonatologists, hepatologists, and transplant surgeons until either hepatic transplantation or liver regeneration takes place [3].

As a result of the detrimental clinical course and prognosis of ALF, liver support devices have been advocated [6] in order to act as bridge to transplantation [3]. Bridging therapy is in even higher demand in cases where transplantation is contraindicated, for example, in patients with consequences of ALF such as uncontrolled sepsis. Liver support devices are promising in ALF due to their inherent capacity of removing accumulated metabolites secondary to deficient hepatic clearance [6].

Extracorporeal liver support (ELS) devices can be divided into bioartificial systems, which use human or animal hepatocytes, and artificial, cell-free systems, as hemadsorption. Currently, there are three types of artificial extracorporeal liver support devices available: Single Pass Albumin Dialysis (SPAD), Molecular Adsorbent Recirculating System (MARS®), and the Fractionated Plasma Separation and Absorption (FPSA), called the “Prometheus System” [7].

Scientific data on ELS concerning the pediatric population is scarce in literature [8, 9]. Although MARS dialysis is the most extensively studied type of ELS, it is limited to case reports and small case series [10].

In the last years, hemadsorption using CytoSorb initially intended as adjunctive treatment for patients with elevated cytokine levels in the setting of SIRS, severe sepsis, and septic shock has gained attention as a potential tool to also reduce various other endogenous and exogenous compounds from blood such as myoglobin, bilirubin, and others. Several publications have shown excellent removal of unconjugated bilirubin (0.6 kDa) and conjugated bilirubin (0.8 kDa) by CytoSorb, with binding confirmed by the beads turning yellow [11, 12]. The CytoSorb polymer is both highly adsorptive and biocompatible, facilitating a concentration-dependent but size-selective removal of molecules with middle molecular weight (approx. 10–50 kDa). Of note, standard high-flux dialysis or CRRT is only capable of removing substances with a molecular weight below 15 kDa.

Herein we present the case of a nine-month-old male infant who was admitted to the NICU due to sepsis after cardiac surgery, Fallot tetralogy and multisystem organ failure (MSOF), respiratory failure, cardiac failure, liver failure, and renal failure which was successfully treated by a combination of continuous hemodiafiltration (HDF) and hemadsorption with a cytokine adsorber (CytoSorb) even despite the lack of an indication and limited information on the use of CytoSorb in children.

## 2. Case Presentation

A nine-month-old male infant, weighing 9 kilograms, was admitted to the NICU of “Marie Curie” Children's Hospital in Bucharest. The patient was admitted from the Pediatric Cardiovascular Surgery Unit, on his fourth postoperative day after the correction of tetralogy of Fallot. The patient was extubated on the first postoperative day, only to be reintubated on the second following the diagnosis of pleural and peritoneal effusions. On the third postoperative day the patient became febrile; therefore a complete blood count (CBC), biochemistry, and a blood culture were performed. The infant entered the NICU in a seriously degraded general status, intubated, and mechanically ventilated (CMV), presenting unilaterally diminished breath sounds, hypoxemia (oxygen saturation: 90%), oliguria, tachycardia (137 beats per minute), hypotension (74/44/59 mmHg), and abdominal distention with anasarca and fever (39-40°C). Physical examination revealed hepatosplenomegaly.

Upon admission, the CBC was reviewed showing hemoconcentration (Hb: 14.2 g/dL, Ht: 45%), thrombocytopenia (27,000/mm^3^), and leukocytosis (17,800/mm^3^) with neutrophilia (66.6%). Coagulation tests revealed a grossly elevated INR (4.66), hypofibrinogenemia (113 mg/dL), and a prolonged aPPT time (63 seconds). From the biochemical tests a severe inflammatory response was noticed (CRP: 47.76 U/L, procalcitonin: 10 ng/L). Furthermore, liver function tests (LFTs) were greatly abnormal (ALT: 1883.1 U/L, AST: 4214.5 U/L, GGT 72 U/L, total bilirubin: 10.05 mg/dL, and direct bilirubin: 7.59 mg/dL), consistent with hepatocellular necrosis. Renal impairment was also evident with creatinine of 1.15 mg/dL and urea of 55 mg/dL. Creatinine kinase and creatine kinase-MB were also elevated with values of 1619 U/L and 159.6 U/L, respectively. Acid-base balance was deranged, revealing metabolic acidosis. Blood culture was sterile and the culture from tracheal aspirate revealed* Escherichia coli* colonies.

Antibiotic and antifungal therapies with Meropenem, Vancomycin, and Fluconazole were initiated. Multiple episodes of severe hypotension and bradycardia were treated with Adrenaline, Noradrenaline, and Dopamine. A central venous catheter was inserted into the left subclavian vein and kept for 34 days. Due to persistence of the systemic inflammatory response syndrome (SIRS) at increasing CRP values from 47.76 to 54.76, Meropenem and Vancomycin were replaced by Tienam (Imipenem and Cilastin) and Amikacin (the dose was adjusted for renal failure) for the duration of 9 days. The metabolic acidosis was managed with sodium bicarbonate. Thrombocytopenia with values as low as 15,000/mm^3^ was treated with multiple transfusions of thrombocyte mass and fresh frozen plasma (FFP). The anasarca was managed with a continuous Furosemide and Aminophylline perfusion and repeated albumin transfusions.

Two days after NICU admission oliguria rapidly evolving to anuria was noticed, and peritoneal dialysis was initiated. While the patient's fluid intake continued to exceed the output the decision was made to start hemodiafiltration (Prismaflex, Baxer) and to insert a double lumen Gambro 6.5 Fr venovenous catheter into the right femoral vein as the access route for HDF. Throughout HDF, the patient continued to be hypotensive and bradycardic necessitating an increased dose of inotropic agents. Metabolic acidosis was also persistent requiring repeated sodium bicarbonate administration. The patient continued to be thrombocytopenic needing multiple thrombocyte mass transfusions. In addition, the infant developed macrocytic, hyperchromic anemia. After initiation of HDF the patient continued to be oliguric presenting intensely hyperchromic hematuria.

The patient's fever did not subside, as a result of which antibiotic therapy was modified once again—this time Tienam and Amikacin were replaced by Meropenem and Ciprofloxacin for a duration of 16 days. Antibiotic doses were kept constant during. The patient's bilirubin levels kept increasing gradually, culminating in a maximum value of 54 mg/dL of total bilirubin and 31.67 mg/dL of direct bilirubin. Intense scleral and cutaneous jaundice was observed and the patient was diagnosed with cholestatic jaundice.

On the 9th day of HDF the therapeutic decision of commencing hemadsorption with a cytokine adsorber (CytoSorb) was taken. Blood flow was maintained at 40 mL/min and anticoagulation was achieved using heparin. The adsorber was placed in postdialyzer position.

Due to the paucity of recommendations in the literature and the fact that a flow of only 40 mL/min—1/5 of the recommended flow for hemadsorption of 200 mL/min—could be maintained throughout the HDF process, the procedure was maintained for 49 hours, 8 times more than normal (6 hours).

**Figure 1:**
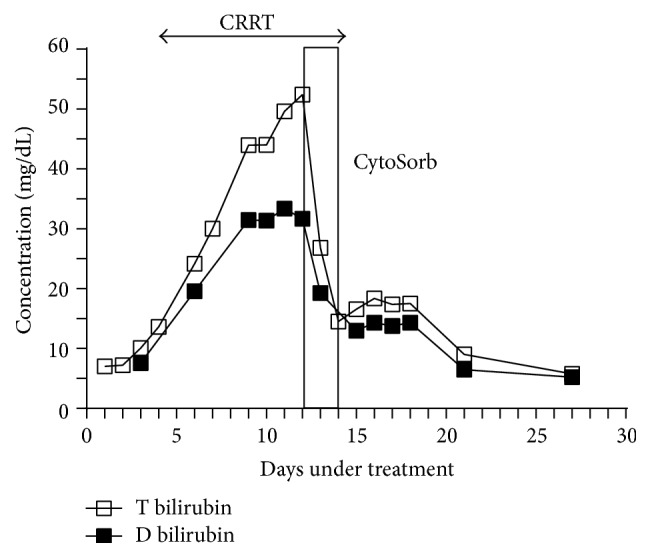
Level of total and direct bilirubin 48 hours before, during, and 48 hours after CytoSorb.

Over the 24-hour period of hemadsorption (during the first half period of the procedure), the total bilirubin value decreased from 54 to 17 mg/dL and the patient's general status improved considerably. Finally, after 49 hours of hemadsorption, the total bilirubin was 14 mg/dL (Figure 1).

**Figure 2:**
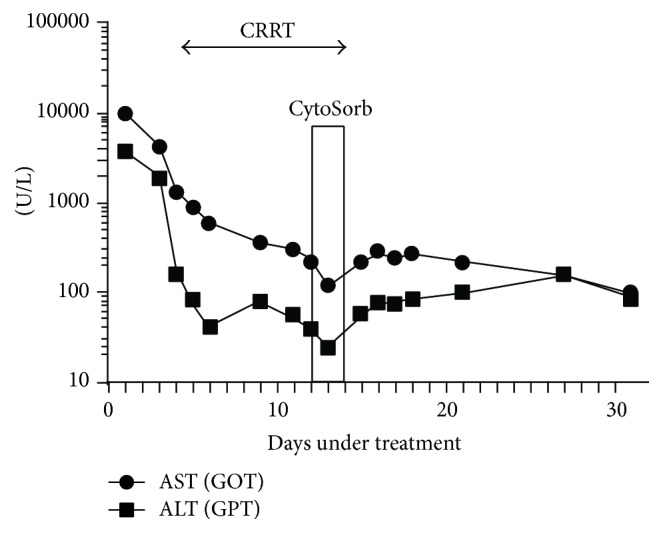
Level of aminotransferases 48 hours before, during, and 48 hours after CytoSorb.

During hemadsorption we noticed a rapid drop of the aminotransferases, which rose back to their previous values as soon as CytoSorb was removed. The infant was discharged three weeks later with aminotransferase values of 95 and 87 U/L for AST and ALT, respectively (Figure 2).

**Figure 3:**
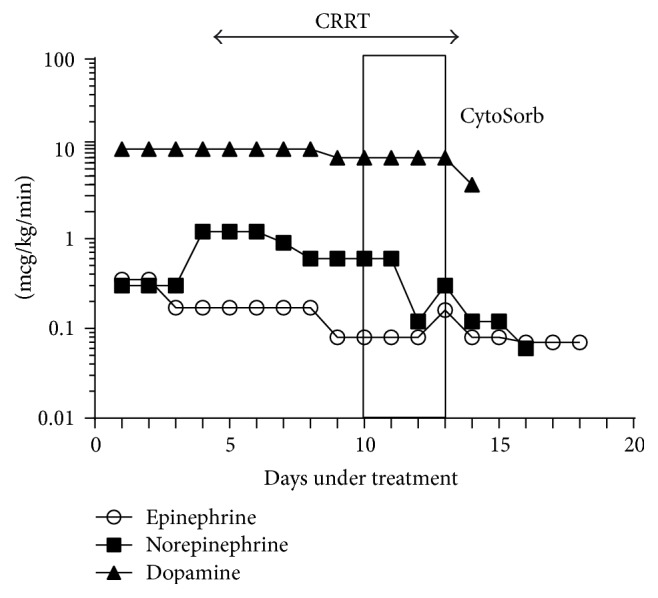
Norepinephrine doses before, during, and 48 hours after CytoSorb.

Our choice of inotropic drugs included norepinephrine, epinephrine, and Dopamine. We were able to decrease the dose rapidly from 0.8 to 0.18 mcg/kg/min during the first day and to stop norepinephrine administration in less than 48 hours from the initiation of CytoSorb treatment. We also managed to decrease the dose from 8 to 5 mcg/kg/min and to finally withdraw Dopamine administration during liver support (6.67 mcg/kg/min represents the medium dose during the CytoSorb treatment) (Figure 3).

The dose of epinephrine was decreased within 48 hours of CytoSorb treatment and withdrawn after 5 days. We observed a slight increase of the dose during CytoSorb treatment. Forty-eight hours after CytoSorb treatment, epinephrine was stopped.

On May 29, HDF was stopped after 11 days. The patient's cardiovascular status improved, which allowed for the dose of the inotropic agents to be decreased. Furthermore, a major attenuation of the SIRS was noticed in addition to a significant improvement of hepatocellular necrosis.

**Figure 4:**
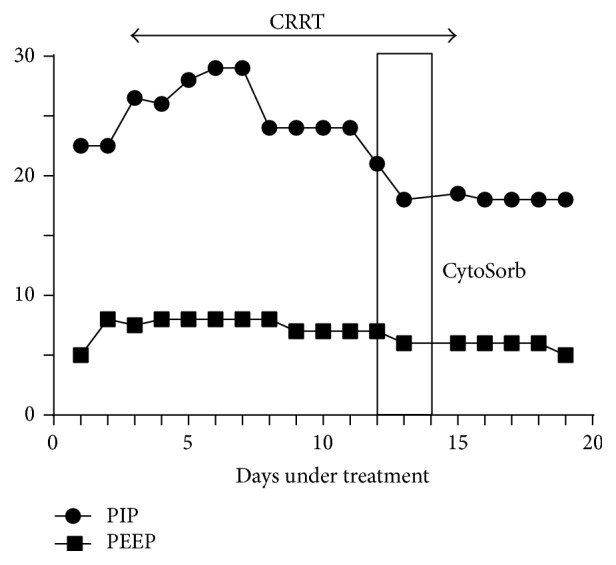
PIP and PEEP settings before, during, and after CytoSorb treatment.

The patient's ventilation settings also improved during CytoSorb treatment. The fraction of inspired oxygen (FiO_2_) dropped from 0.4 to 0.3 in the first day and subsequently to room air on the third day of liver treatment (at 49 hours). The respiratory rate (RR) decreased rapidly from 29 to 22 breaths/minute during the first day and then to 20 breaths/minute and maintained there for five days until extubation. Positive inspiratory pressure (PIP) decreased from 24 to 18 cm/H_2_O while positive end expiratory pressure (PEEP) decreased from 7 to 6 cm/H_2_O for the last five days on mechanical ventilation until extubation, having totally 27 days of mechanical ventilation (Figure 4).

Diuresis gradually reached normal values and on the same day enteral nutrition was initiated.

Antibiotic doses could also be kept constant during the entire hemadsorption procedure using CytoSorb.

Renal function returned to normal with normochromic urine in a normal quantity while renal function tests showed normalization of the values of serum creatinine and urea.

The patient was discharged home on June 18, after 34 days of hospitalization, in a good general status, hemodynamically stable, afebrile, and weighing 9.6 kilograms. The patient's blood tests upon discharge were as follows: CBC within normal limits, mild inflammatory syndrome (slight leukocytosis (11,500/mm^3^), CRP 5.27 mg/dL), hyperbilirubinemia (total: 5.89 mg/dL, conjugated: 5.24 mg/dL), increased transaminases (ALT: 87 U/L, AST: 95 U/L), normal serum creatinine, urea, and electrolytes. He was referred as well to pediatric neurologist for follow-up.

## 3. Discussion

Despite progress in critical care and highly specialized intensive care units, ALF is still a multisystem condition associated with high mortality [3, 13]. Artificial extracorporeal liver support devices were designed to improve the survival of patients with ALF by carrying out the detoxification processes, in the form of bridge therapy, while awaiting transplantation [14].

Hemadsorption has proven to be a safe and efficacious modality of bridging therapy in adults for treating complications of liver failure such as hepatic encephalopathy (HE), hemodynamic instability, and progressive hyperbilirubinemia [6].

The scientific data about ELS regarding the pediatric population is scarce. The largest study on the pediatric population thus far is coming from a hospital with ten years of experience with the MARS method. In the study performed by Lexmond et al., MARS was employed as ELS in 20 pediatric patients suffering from ALF listed for high urgency liver transplantation. The decision to put the children on MARS was made by a multidisciplinary team consisting of pediatricians, hepatologists, nephrologists, neurologists, and transplant surgeons. MARS was applied for eight consecutive hours daily, every day, up until transplantation. All patients were mechanically ventilated and on vasopressors [10], as was the case in our patient. This method decreased serum ammonia and bilirubin [10]. The only encountered adverse effect from the therapy in this study was thrombocytopenia with bleeding requiring blood and platelet mass transfusions in five children, with one case of mortality [10].

The first particularity of this case was the extreme condition of the infant suffering from sepsis with MSOF, with intravascular hemolysis and thrombocytopenia reaching a value of 15.000/mm^3^. Next, the etiology of the severe coagulopathy was questionable; it could have been attributable solely to ALF, but also to sepsis, or a combination of both. Also, the jaundice could have been a result of ALF, but also of TPN. Parenteral nutrition-associated cholestasis (PNAC) is one of the most common complications of TPN in neonates and infants. In a study performed by Hsieh et al. in 2009 on 62 infants on TPN, 11 developed PNAC [15]. Furthermore, another particularity that arose was the degree of association between congenital heart disease and ALF. A link between vascular malformations and congenital heart disease with ALF has been described in literature [4, 5]. Another particularity of the case was the advanced degree of hemodynamic instability with hypotension present despite a triple regimen of vasopressors (Adrenaline, Noradrenaline, and Dopamine).

Progressive liver failure can result in death within a few days if no liver is transplanted [16]. We performed hemadsorption on a nine-month-old male infant as a worldwide novel procedure and obtained favorable results. Despite the extreme general status of our intubated patient requiring triple vasopressor therapy, at total bilirubinemia of 54 mg/dL, 24 hours of hemadsorption deemed to be successful. However, since literature regarding hemadsorption in the pediatric population is scarce, large scale prospective studies are needed to determine the exact benefit and possible adverse effects as well as assess survival rates after such a procedure.
